# Chlorogenic Acid from *Peucedanum japonicum* Attenuates TNF-α-Induced Oxidative Stress and Inflammatory Damage in Human Dermal Fibroblasts

**DOI:** 10.3390/life15121934

**Published:** 2025-12-18

**Authors:** Neil Patrick Uy, Minseo Kang, Jang Hoon Kim, Young Ho Hoon, Sanghyun Lee, Sullim Lee

**Affiliations:** 1 Department of Plant Science and Technology, Chung-Ang University, Anseong 17546, Republic of Korea; uyneilpatrick@gmail.com; 2 Department of Life Science, College of Bio-Nano Technology, Gachon University, Seongnam 13120, Republic of Korea; bana825@gachon.ac.kr; 3 Department of Herbal Crop Research, National Institute of Horticultural and Herbal Science, Rural Development Administration, Eumseong 27709, Republic of Korea; jhkim53@korea.kr (J.H.K.); yhyoon@korea.kr (Y.H.H.); 4 Natural Product Institute of Science and Technology, Anseong 17546, Republic of Korea

**Keywords:** skin aging, reactive oxygen species, MMP-1, secondary metabolites, inflammation

## Abstract

Intrinsic aging and external stimuli such as UV exposure contribute to heightened MMP-1 expression, leading to collagen deterioration and weakening of the skin’s structural framework, hallmarks of aging tissue. *Peucedanum japonicum*, a plant consumed in East Asia, contains antioxidant and anti-inflammatory compounds, but its effects on skin aging remain unclear. This study profiled six major bioactive compounds in *P. japonicum* leaves and roots and evaluated their protective effects in TNF-α-stimulated human dermal fibroblasts (NHDFs). Phytochemical profiles were determined, and biological activity was evaluated by measuring intracellular ROS, MMP-1 secretion, and COL1A1 expression. Both leaf and root extracts exhibited antioxidant and anti-inflammatory activity, with leaves generally showing stronger effects. Among the six compounds, chlorogenic acid (**1**) demonstrated the most potent activity. It markedly decreased intracellular ROS, suppressed MMP-1 secretion, and enhanced COL1A1 expression in TNF-α-stimulated NHDFs, indicating protection against inflammation-induced collagen degradation. These findings suggest that *P. japonicum*, particularly its chlorogenic acid (**1**) content, may be a promising natural resource for anti-aging skincare and therapies targeting inflammation-associated skin damage.

## 1. Introduction

Skin aging is primarily driven by intrinsic and extrinsic factors and results from a variety of biological processes and complex interactions. Among these, extrinsic aging is mainly attributed to environmental stressors such as ultraviolet (UV) radiation, air pollution, and chemical exposure. This form of aging is strongly associated with the excessive production of reactive oxygen species (ROS) and the sustained activation of inflammatory signaling pathways [1].

Oxidative stress triggered by ROS leads to the deterioration of vital cellular structures such as lipids, proteins, and DNA, and enhances the expression of matrix metalloproteinases (MMPs), which in turn contribute to the breakdown of extracellular matrix (ECM) components. In particular, MMP-1 selectively degrades type I collagen, the major structural protein found in the dermal layer of the skin [2,3]. Elevated MMP-1 activity speeds up the degradation of collagen, which manifests as common aging indicators like wrinkle development and diminished skin elasticity. Furthermore, tumor necrosis factor-alpha (TNF-α), a major pro-inflammatory cytokine rapidly induced by UV radiation, amplifies oxidative and inflammatory responses by promoting ROS generation and upregulating MMP expression, ultimately contributing to extracellular matrix (ECM) degradation and structural skin damage. TNF-α also enhances the production of additional inflammatory mediators, including interleukin (IL)-1β, IL-8, and IL-6, thereby intensifying cutaneous inflammation [4,5]. In addition, TNF-α activates the mitogen-activated protein kinase (MAPK) pathway through phosphorylation, leading to increased MMP expression, accelerated collagen breakdown, and progression of skin aging.

In recent years, increasing attention has been directed toward the identification of natural bioactive compounds that are safe and exhibit anti-aging, anti-inflammatory, and antioxidant properties. Among these, plant-derived compounds such as polyphenols and coumarins have demonstrated remarkable therapeutic potential by regulating oxidative stress, suppressing inflammation, and enhancing collagen synthesis [6,7,8,9].

*Peucedanum japonicum* Thunb., a perennial species of the Apiaceae family, is primarily found across East Asia and has a long-standing history of use as a medicinal and dietary plant in Korea and Japan. In Korea, it is commonly referred to as “Sikbangpung” or “Gaetgireumnamul”. Traditionally, both the leaves and roots of *P. japonicum* have been incorporated into herbal remedies aimed at relieving inflammatory conditions, such as cough, bronchitis, and minor skin injuries [10,11]. This plant is a rich source of the aforementioned bioactive molecules [12].

In this study, phytochemical profiling was conducted to quantify and compare the physiologically active components present in various organs of plants. Subsequently, the effects of crude extracts and six compounds obtained from *P. japonicum* were assessed on ROS production, MMP-1 production, and procollagen type I α1 (COL1A1) expression in TNF-α-treated NHDFs [13]. The most effective compound was selected, and its effect on the phosphorylation of the MAPK pathway and mRNA expression of inflammatory cytokines was further confirmed. Although *P. japonicum* is not traditionally recognized for its anti-aging properties, its historical applications provide a rationale for investigating the ability of this plant to alleviate oxidation-derived stress and reduce inflammation-driven damage in dermal fibroblasts [14].

## 2. Materials and Methods

### 2.1. Plant Sample Acquisition and Handling

*P. japonicum* samples were cultivated in Yeosu, South Korea, and harvested at multiple time points in 2021 by the Department of Herbal Crop Research, NIHH, RDA. Each sample code was designated as follows: YML, YAL, and YNL refer to *P. japonicum* leaf (L) extracts harvested in March (M), April (A), and November (N), respectively, from Yeosu (Y), Korea, while YMR, YAR, and YNR correspond to root (R) extracts collected in the same periods.

### 2.2. Chemicals and Reagents

**Figure 1:**
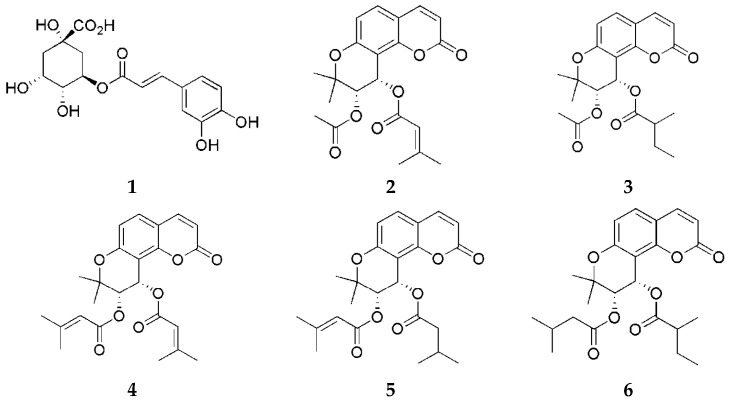
Chemical structures of compounds **1**–**6**.

For extraction procedures, 95% ethanol was sourced from Samchun Chemicals (Pyeongtaek, Republic of Korea). The solvents used for high-performance liquid chromatography (HPLC), including methanol, acetonitrile, and ultrapure water, were purchased from Honeywell Burdick & Jackson (Muskegon, MI, USA). Formic acid was obtained from Thermo Fisher Scientific (Waltham, MA, USA). Reference standards were previously isolated and structurally identified from *P. japonicum* [13,14] (Figure 1).

### 2.3. Sample Extraction

Each plant sample (10 g) underwent three cycles of reflux extraction with 95% EtOH as the solvent, with each extraction performed for 5 h under controlled heating. Following every extraction, the mixture was filtered, and the combined filtrates were evaporated under reduced pressure at 60 °C using a rotary evaporator (Eyela, Tokyo, Japan) until complete solvent removal was achieved.

### 2.4. Chromatographic Conditions

Stock solutions of compounds **1**–**6** were prepared by dissolving 1 mg of each compound in 1 mL of MeOH (1 mg/mL). Extracts were prepared at 30 mg/mL with the same solvent. Standard and sample solutions underwent 15 min of sonication and were subsequently filtered with a 0.45 µm PVDF membrane. Chromatographic separations were performed using a gradient elution with solvent A (0.1% formic acid in water) and solvent B (ACN), beginning with 90% solvent A for 10 min, decreasing to 0% solvent A from 25 to 30 min, and finishing with 10% B held from 31 to 40 min. The mobile phase flow rate was 1.0 mL/min, with a 10 µL injection volume and detection at 320 nm. The method was validated by assessing the limits of detection and quantification for all analytes [13].

### 2.5. Calibration Curves

Calibration curves were generated from the standard solution to quantify the content of each compound in the extracts. Each curve was obtained by plotting peak area (Y) versus standard concentration (X, μg/mL) to derive the calibration equations. The results are expressed as mean ± standard deviation (*n* = 3).

### 2.6. Evaluation of Cytotoxicity

NHDFs were dispensed onto clear-bottom 96-well plates at a density of 1 × 10^4^ cells/well and incubated for 24 h to allow adherence. Subsequently, the cells were exposed to different concentrations of the plant extracts and compounds **1**–**6**. After further incubation for 24 h under standard conditions, cell viability was evaluated by measuring the metabolic activity using the EZ-Cytox reagent (DoGenBio Co., Ltd., Seoul, Republic of Korea). Cell viability was measured by reading the absorbance at 450 nm with a microplate spectrophotometer.

### 2.7. Evaluation of Intracellular ROS Accumulation

Cells were seeded into 96-well plates at a density of 1 × 10^4^ cells/well and allowed to adhere for 24 h. The growth medium was then exchanged for serum-free medium for an additional 24 h to create nutrient-deprivation conditions. After this period, the cells were treated with the plant extracts or isolated compounds for 1 h before being challenged with TNF-α (20 ng mL^−1^) for 15 min. Intracellular ROS production was assessed by incubating the cells with 2′,7′-dichlorodihydrofluorescein diacetate (DCFDA) for 15 min, followed by a single PBS wash and fluorescence measurement at 485/530 nm using an INNO plate reader (LTek Co., Ltd., Seongnam, Republic of Korea). For visual analysis, NHDFs were seeded at 2 × 10^4^ cells per well in 48-well plates and subjected to the same treatment regimen, after which DCFDA-stained cells were imaged to evaluate ROS distribution.

### 2.8. Quantification of Secreted Proteins by Enzyme-Linked Immunosorbent Assay (ELISA)

For the determination of secreted protein levels, NHDFs were plated in 48-well plates at 1 × 10^4^ cells per well and incubated for 24 h, followed by serum deprivation for another 24 h. After pretreatment with the samples for 1 h, cells were challenged with TNF-α (20 ng/mL) and maintained for 24 h. The resulting supernatants were collected and analyzed using commercial ELISA kits (c-LEcta GmbH, Leipzig, Germany) to measure MMP-1 and COL1A1 concentrations. Optical density readings were recorded at 450 nm on a microplate reader to quantify secreted protein levels.

### 2.9. Protein Expression Analysis by Western Blotting

For immunoblot analysis, NHDFs were seeded into 6-well plates at 3 × 10^5^ cells per well and cultured for 24 h, after which serum was removed for another 24 h. The cultures were treated with chlorogenic acid (**1**) for 1 h before exposure to TNF-α (20 ng/mL) for 15 min. Total proteins were extracted using RIPA lysis buffer and quantified with a bicinchoninic acid assay (Merck, Rahway, NJ, USA). Samples were electrophoresed using SDS-PAGE, transferred onto membranes, and probed with antibodies recognizing phosphorylated ERK, p38, and JNK, along with GAPDH as the internal reference (Cell Signaling Technology, Danvers, MA, USA). Primary antibody incubation was performed overnight at 4 °C, followed by HRP-linked secondary antibody treatment for 1 h. Signal development utilized SuperSignal™ West Femto chemiluminescent reagent (Thermo Fisher Scientific, Waltham, MA, USA), and bands were visualized using a Fusion Solo imaging system (PEQLAB Biotechnologie GmbH, Erlangen, Germany) [15].

To allow direct comparison of phosphorylated and total forms of each MAPK protein, membranes subjected to phosphoprotein detection were subsequently processed using a standardized stripping procedure. Membranes were incubated in stripping buffer for approximately 2 h, thoroughly washed, and then reprobed with the corresponding total protein antibodies under identical detection conditions. This stripping–reprobing workflow was applied consistently to ERK, JNK, and p38, enabling normalization of each phosphoprotein to its respective total protein within the same membrane.

### 2.10. Real-Time PCR

NHDFs were plated in 6-well plates at 3 × 10^5^ cells per well and incubated for 24 h, followed by a serum-free culture period of 24 h. Cells were then treated with compound 1 for 1 h and stimulated with TNF-α (20 ng/mL) for 4 h. Total RNA was isolated using the RNeasy Mini Kit (QIAGEN, Hilden, Germany), and cDNA was synthesized with the RevertAid First-Strand cDNA Synthesis Kit (Thermoscientific, Waltham, MA, USA). Quantitative PCR analysis of IL-6, IL-8, and GAPDH transcripts was carried out using TOPreal™ SYBR Green qPCR High-ROX Premix (Enzynomics, Daejeon, Republic of Korea) [16].

### 2.11. Visualizing Anti-Aging Efficacy Using Radar Charts

The range of each metric was split into four zones based on its lowest and highest values, and every concentration tier received a matching score. A radar chart was created to visualize and check the mean values for each concentration group. The area surrounded by lines connecting the data points reflects the relative efficacy of the treatment [17].

### 2.12. Statistical Analysis

The data are expressed as mean ± SD for HPLC measurements and as mean ± SEM for the cell-based assays. Differences among groups were assessed with one-way ANOVA, followed by Tukey’s post hoc test. All statistical calculations were carried out in GraphPad Prism (v5.0, GraphPad Software, Boston, MA, USA). A *p*-value of less than 0.05 was considered statistically significant.

## 3. Results

### 3.1. Crude Extract Bioactivity Screening

All *P. japonicum* extracts were screened for their effects on NHDF viability. Among them, YMR, YNR, and YAR exhibited mild cytotoxicity at the highest tested concentration (50 μg/mL), whereas YML, YAL, and YNL showed no cytotoxic effects at any concentration. In the intracellular ROS assay, all extracts significantly reduced TNF-α-induced ROS accumulation in NHDFs at all tested concentrations, except for YMR, which consistently exhibited a notably weaker antioxidant effect. Therefore, further analyses were conducted to identify the phytochemical constituents in each extract.

### 3.2. Quantitative Analysis of Compounds ***1**–**6***

**Figure 2:**
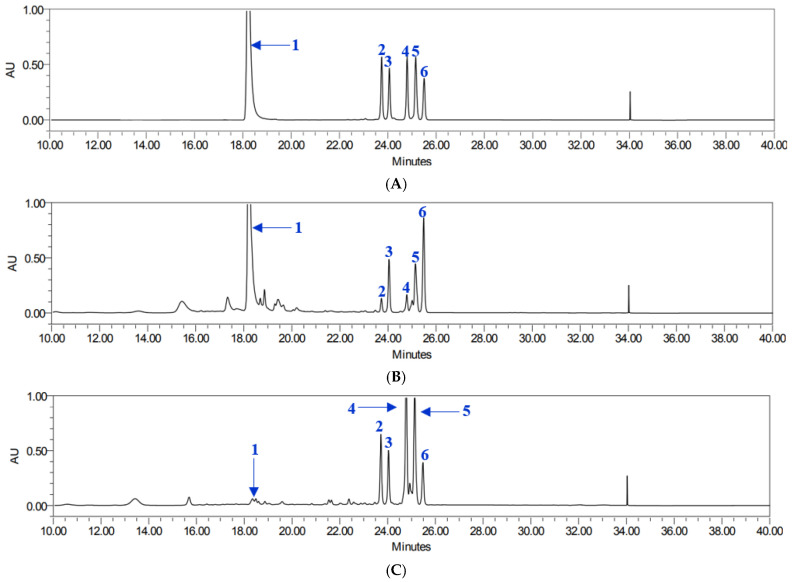
HPLC chromatogram of compounds **1**–**6** (**A**), YML (**B**), and YNR (**C**).

All analytes demonstrated excellent linearity, with coefficients of determination (*R*^2^) ranging from 0.9996 to 1.0000. Retention times ranged from 18.25 to 25.42 min. Using this validated method, the contents of six target compounds were quantified in both leaf and root extracts (Table 1), showing good chromatographic separation and retention times (Figure 2). The total compound content varied depending on the plant part. Compound **1** was most abundant in leaf extracts, with particularly high levels in YML and YAL, whereas root extracts contained considerably lower amounts. Pyranocoumarins tended to accumulate more in roots than in leaves. Specifically, compounds **5** and **4** reached their highest concentrations in YNR. Compounds **2** and **3** also followed a root-preferential distribution pattern, with compound **3** being most abundant in YAR and compound **2** found in relatively high levels in both YAR and YMR. In contrast, compound **6** was present at comparatively high levels in all leaf extracts, particularly in YAL and YML, while root extracts exhibited lower concentrations, with the lowest levels observed in YNR.

### 3.3. Effects of the Extracts and Compounds ***1**–**6*** on NHDF Cell Viability

**Figure 3:**
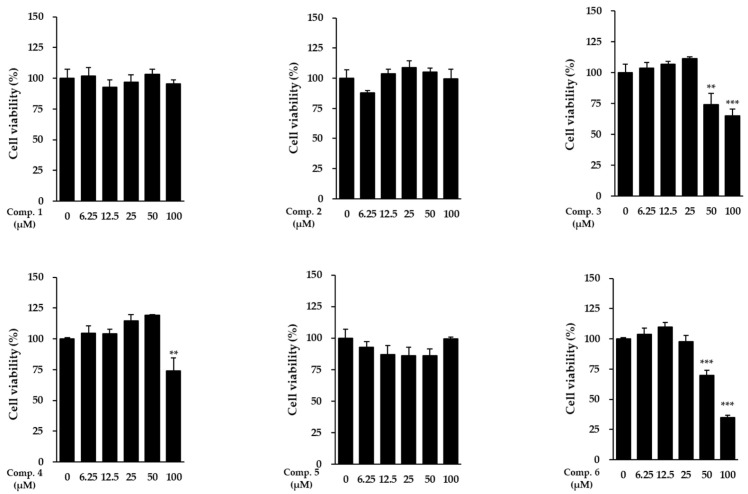
Cell viability of NHDFs after exposure to compounds **1**–**6**. Values represent mean ± SEM from triplicate experiments. Statistical notation: ** *p* < 0.01 and *** *p* < 0.001 compared with vehicle-treated cells.

Cell viability was assessed to establish safe working concentrations. Each compound was applied to NHDFs at predetermined doses, and cell viability was subsequently measured (Figure 3). Notably, compound **1** exhibited no cytotoxicity across the entire concentration range tested, including at the highest dose of 100 μM, indicating that it is well tolerated by dermal fibroblasts within this range. Similarly, compounds **4** and **5** showed no detectable cytotoxicity at any concentration. In contrast, compound **3** displayed cytotoxicity at 100 μM, while compounds **2** and **6** exhibited cytotoxic effects at 50 μM. Accordingly, only non-cytotoxic concentrations (≤100 μM for compound **1** and lower thresholds for the other compounds) were used in subsequent experiments.

### 3.4. Effects of Compounds ***1**–**6*** on Intracellular ROS Secretion in TNF-α-Stimulated NHDFs

**Figure 4:**
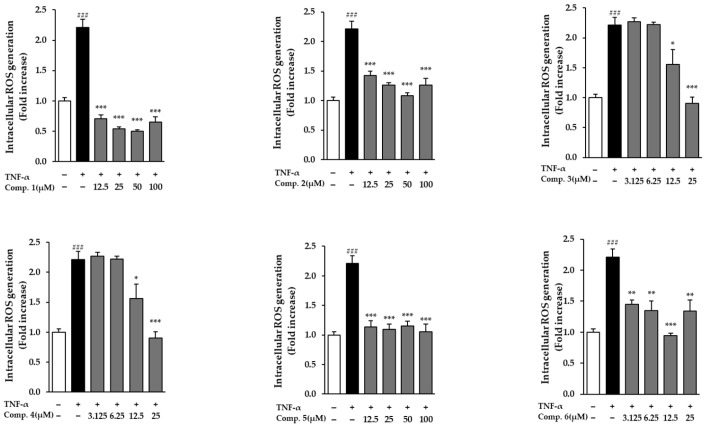
ROS levels in TNF-α-stimulated NHDFs pretreated with compounds **1**–**6**. Data reflect three independent replicates (mean ± SEM). Statistical comparisons: ### *p* < 0.001 vs. vehicle; * *p* < 0.05, ** *p* < 0.01, *** *p* < 0.001 vs. TNF-α alone.

**Figure 5:**
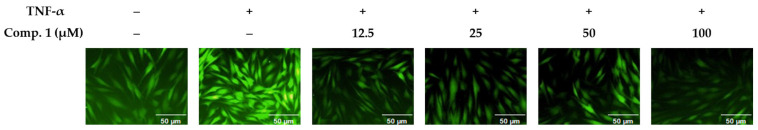
Fluorescence microscopy images illustrating ROS accumulation in TNF-α-stimulated NHDFs and the suppressive effect of compound **1**.

In NHDFs activated with TNF-α, all evaluated drugs dramatically decreased ROS levels (*p* < 0.05) (Figure 4). A significant increase in ROS generation was brought on by TNF-α exposure, suggesting the activation of oxidative stress. Treatment with the compounds, however, notably mitigated this elevation relative to the TNF-α-only control group. Notably, compound **1** demonstrated a dose-dependent reduction in ROS levels, as indicated by decreased fluorescence intensity in TNF-α-treated cells (Figure 5). These results suggest that compound **1** effectively mitigates oxidative stress and may serve as a promising phytotherapeutic agent for preventing ROS-related skin damage, which is often exacerbated by environmental stressors such as UV exposure.

### 3.5. Effects of Compounds ***1**–**6*** on MMP-1 and COL1A1 Protein Secretion in TNF-α-Stimulated NHDFs

**Figure 6:**
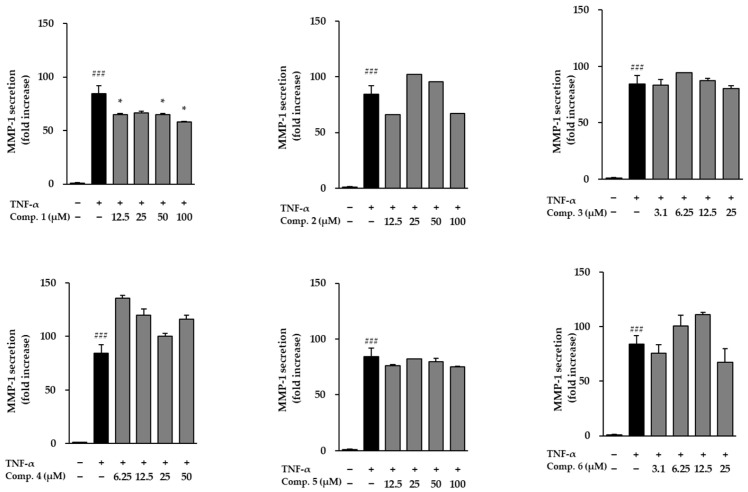
Modulation of MMP-1 release by compounds **1**–**6** in TNF-α-activated NHDFs. MMP-1 secretion in culture media was quantified by ELISA and expressed as fold change relative to untreated cells. Bars represent mean ± SEM from duplicate datasets. ### *p* < 0.001 vs. control; * *p* < 0.05 vs. TNF-α-treated cells.

**Figure 7:**
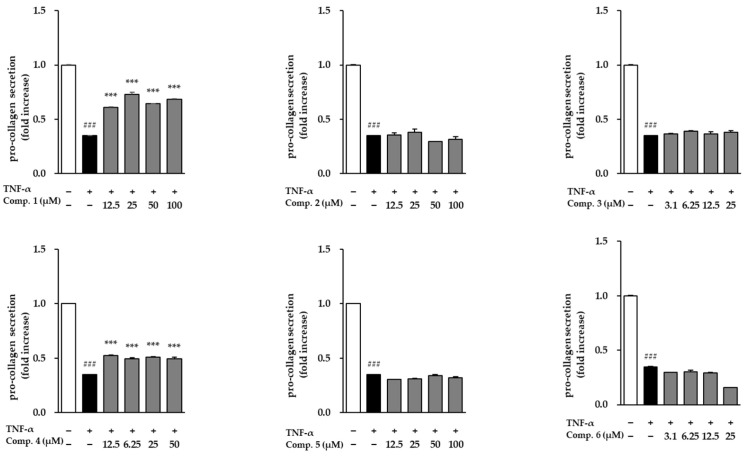
The modulation of COL1A1 secretion by compounds **1**–**6** in TNF-α-challenged NHDFs. COL1A1 levels were determined by ELISA and reported as fold difference relative to vehicle-treated groups (mean ± SEM; *n* = 2). ### *p* < 0.001 vs. control; *** *p* < 0.001 vs. TNF-α.

Following stimulation with TNF-α, NHDFs exhibited a dramatic surge in MMP-1 secretion, reaching 84.30 ± 7.83-fold above baseline, which reflects a substantial increase in matrix breakdown and tissue deterioration (Figure 6). When the six isolated compounds were assessed for their ability to modulate this response, only compound **1** was able to counteract the TNF-α-induced rise in MMP-1 levels. The remaining compounds did not noticeably alter MMP-1 secretion under the same conditions. These findings suggest that compound **1** may exert anti-aging and anti-inflammatory effects by inhibiting extracellular matrix degradation. The influence of compounds **1**–**6** on COL1A1 secretion in TNF-α-stimulated NHDFs is presented in Figure 7. TNF-α, respectively, reduced procollagen levels (0.35 ± 0.00-fold, *p* < 0.001), indicating impaired collagen synthesis. However, treatment with compounds **1** and **3** significantly restored procollagen secretion, suggesting their potential roles in promoting collagen synthesis and counteracting TNF-α-induced skin aging.

### 3.6. Radar Chart Evaluation of Compounds ***1**–**6*** in TNF-α-Stimulated NHDFs

**Figure 8:**
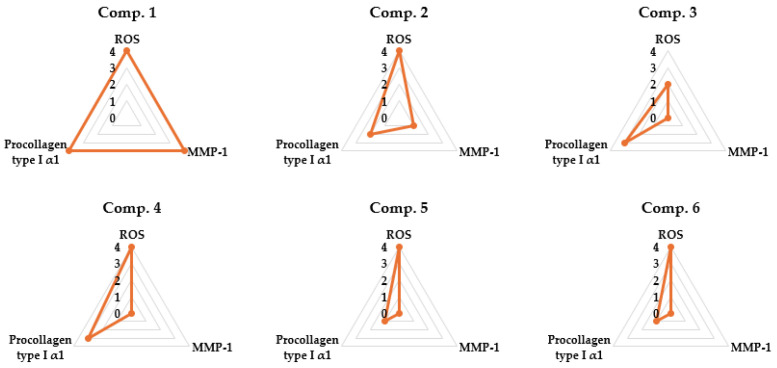
Radar chart summarizing anti-aging-related outcomes for compounds **1**–**6** across three biological endpoints: ROS suppression, MMP-1 inhibition, and COL1A1 restoration. Data were normalized to provide a relative comparative assessment of overall protective activity.

A set of radar charts depicts the relative effects of compounds **1**–**6** on TNF-α–stimulated NHDFs, focusing on three outcome measures: ROS production, COL1A1 release, and MMP-1 levels (Figure 8). Among the six compounds evaluated, compound **1** exhibited the most pronounced multi-target efficacy by simultaneously mitigating oxidative stress, promoting collagen synthesis, and suppressing extracellular matrix breakdown. These findings highlight the potential of compound **1** as a strong candidate for anti-aging skincare applications by targeting multiple cellular mechanisms.

### 3.7. Effect of Chlorogenic Acid *(**1**)* on MAPK Phosphorylation in TNF-α-Stimulated NHDFs

**Figure 9:**
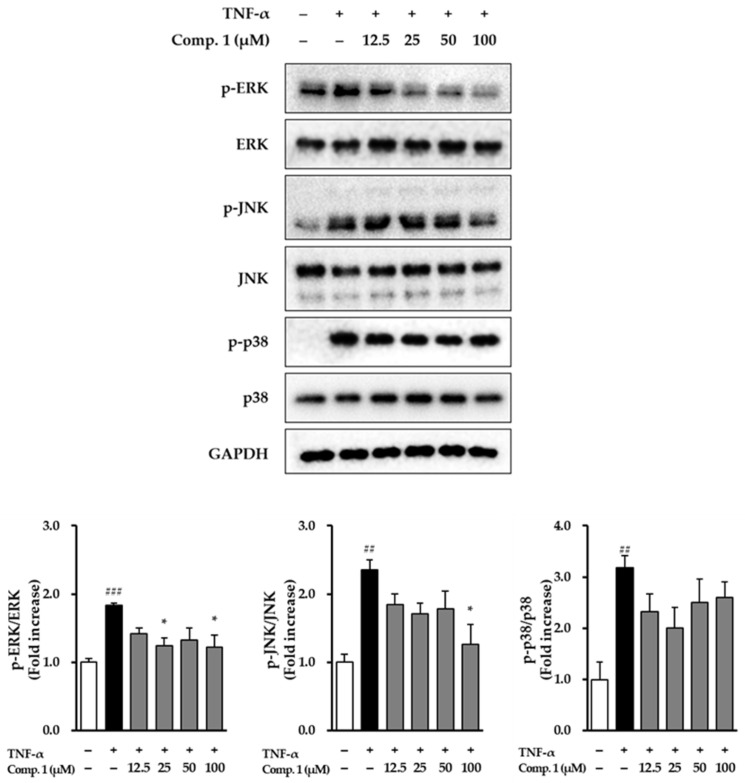
MAPK phosphorylation pathways affected by compound **1** in NHDFs after TNF-α stimulation. Cells were preincubated with compound **1** for 1 h and then stimulated with TNF-α (20 ng/mL) for 15 min. Expression levels of phosphorylated and total JNK, ERK, and p38 were analyzed by immunoblotting, using GAPDH as a reference. Phosphoprotein signals were detected first, after which the same membranes were stripped and reprobed to detect the corresponding total proteins. Fold change values (mean ± SEM; *n* = 3) were calculated relative to controls. ## *p <* 0.01, and ### *p* < 0.001 vs. vehicle control; * *p* < 0.05 vs. TNF-α-stimulated group.

To explore how chlorogenic acid (**1**) protects NHDFs, its effects on the MAPK signaling pathway under TNF-α stimulation were examined (Figure 9). Chlorogenic acid (**1**) treatment counteracted TNF-α–induced MAPK activation, with distinct effects observed across the ERK, JNK, and p38 pathways. For ERK, TNF-α stimulation increased phosphorylation to 1.83 ± 0.04-fold (*p* < 0.001) relative to untreated cells. Chlorogenic acid (**1**) produced modest reductions at lower concentrations and a significant decrease at 100 μM (1.22 ± 0.18, *p* < 0.05). For JNK, TNF-α elevated phosphorylation to 2.35 ± 0.15-fold (*p* < 0.01). Treatment with chlorogenic acid (**1**) markedly reduced this activation at 100 μM, lowering the level to 1.27 ± 0.29 (*p* < 0.05). For p38, TNF-α induced the most pronounced activation, increasing phosphorylation to 3.18 ± 0.23-fold (*p* < 0.01). Chlorogenic acid (**1**) moderately attenuated this increase, particularly at 25 μM (2.01-fold). Collectively, these findings indicate that compound **1** effectively mitigates TNF-α–induced activation of ERK, JNK, and p38 MAPKs, with especially strong inhibition of ERK. These results suggest that the anti-inflammatory activity of compound **1** may, at least in part, be mediated through suppression of these critical MAPK pathways in NHDFs.

### 3.8. Effects of Chlorogenic Acid *(**1**)* on Pro-Inflammatory Cytokines in TNF-α-Stimulated NHDFs

**Figure 10:**
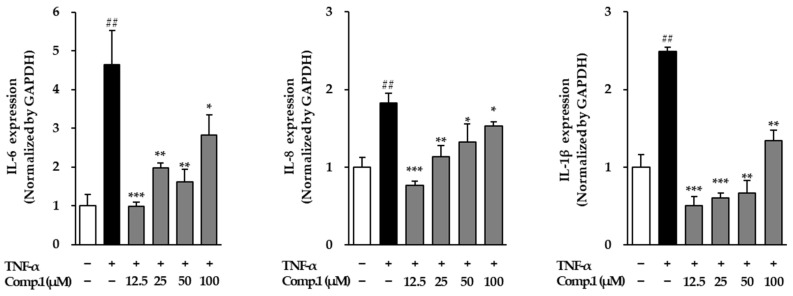
Gene expressions of the inflammatory mediators post-treatment with compound **1** in TNF-α-stimulated NHDFs. Cells were pretreated with compound **1** for 1 h prior to TNF-α exposure for 4 h. Cytokine mRNA levels were determined via RT-PCR and normalized to GAPDH, then expressed as fold changes relative to untreated cells (mean ± SEM, *n* = 3). ## *p* < 0.01 vs. vehicle control; * *p* < 0.05, ** *p* < 0.01, and *** *p* < 0.001 vs. TNF-α-stimulated group.

To further explore the anti-inflammatory potential of chlorogenic acid (**1**), we examined its effect on the mRNA expression of major pro-inflammatory cytokines, including IL-6, IL-8, and IL-1β, in TNF-α-stimulated NHDFs (Figure 10). TNF-α stimulation triggered a robust inflammatory response, as evidenced by a 4.64-fold increase in IL-6, a 1.83-fold rise in IL-8, and a 2.49-fold elevation in IL-1β relative to untreated cells. Chlorogenic acid (**1**) treatment significantly modulated this response in a dose-dependent manner. IL-6 expression decreased at the lowest concentration (0.98-fold at 12.5 μM), with moderate suppression at 25 μM (1.97-fold) and 50 μM (1.62-fold), and a partial recovery at the highest dose (2.82-fold at 100 μM). Similarly, IL-8 levels were reduced to 0.76-, 1.13-, 1.32-, and 1.53-fold at 12.5, 25, 50, and 100 μM, respectively. IL-1β followed a comparable pattern, showing notable downregulation to 0.51-, 0.61-, 0.67-, and 1.34-fold in the same set of concentrations. These results highlight the capacity of compound 1 to dampen TNF-α-induced cytokine production, suggesting that it exerts strong anti-inflammatory effects by modulating key signaling pathways involved in the inflammatory cascade.

## 4. Discussion

The skin is made up of the epidermis and dermis, with the dermis providing structural support through its rich content of ECM, notably collagen. The degradation of these structures during aging leads to wrinkles and reduced elasticity. Both intrinsic factors (genetic programming) and extrinsic factors, such as UV radiation, contribute to this process [18]. Among extrinsic stressors, UV irradiation is a major driver of photoaging, primarily by inducing excessive ROS that activate redox-sensitive inflammatory and degradative pathways [19,20]. Elevated ROS levels damage cellular macromolecules and accelerate the breakdown of ECM proteins, including collagen and elastin, while upregulating MMPs, particularly MMP-1, which selectively degrades type I collagen [21,22]. In parallel, UV-induced cytokines, such as TNF-α, further amplify ROS production and stimulate inflammatory mediators (IL-6, IL-1β, and IL-8), contributing to chronic inflammation and dermal structural decline [23].

Given the limitations of synthetic compounds and the increasing interest in natural alternatives, numerous plant-derived substances, particularly polyphenols, flavonoids, and coumarins, have gained attention for their ability to alleviate inflammation and collagen-preserving activities [24,25]. These bioactive compounds modulate the signaling pathways associated with ECM remodeling and have been widely explored as potential anti-aging agents.

To identify potential plant-based interventions targeting skin aging mechanisms, HPLC-based detailed profiling and quantification of major plant compounds in *P. japonicum* extracts were performed. Compound **1** was identified as the major component in leaves, consistent with its well-known role as a key polyphenol associated with UV protection, oxidative stress mitigation, and defense against herbivores [14,26]. These protective functions are particularly important in photosynthetically active aboveground tissues exposed to environmental stress factors. In contrast, coumarin derivatives were primarily detected in the roots, where they are typically synthesized and stored. These compounds may act as plant allelochemicals, antimicrobial agents, or regulators of root-soil interactions [27], reflecting the plant’s adaptation to underground biological stress factors, such as pathogenic microorganisms and allelopathic competition.

The perennial herb *P. japonicum*, native to East Asia, has long been utilized in traditional medicine and as a food ingredient in Korea. This plant contains various bioactive components, including coumarins and compound **1**, known for their antioxidant, anti-inflammatory, and liver-protective effects [28,29]. However, its protective effect against skin cell damage caused by TNF-α is not well reported.

This study aimed to assess the anti-aging properties of *P. japonicum* extracts and six isolated compounds using TNF-α-stimulated NHDFs as a model. Differences in their cytotoxicity and antioxidant capacity were largely attributable to the distinct phytochemical composition of each extract, highlighting the importance of specific bioactive constituents in mediating cellular protection. Leaf extracts rich in compound **1** showed no cytotoxicity, whereas root extracts, containing higher levels of pyranocoumarins, exhibited mild cytotoxicity and weaker bioactivity, suggesting that excessive coumarins may impair fibroblast viability.

All tested compounds reduced TNF-α-induced ROS levels; however, their effects on MMP-1 and COL1A1 expression differed. Compound **1** uniquely downregulated MMP-1 and, together with compound **3**, restored COL1A1 synthesis, indicating the most comprehensive protective activity through simultaneous suppression of oxidative stress and support of collagen homeostasis. Although compound **4** enhanced COL1A1 expression without reducing MMP-1 expression, this is consistent with prior findings showing that type I procollagen synthesis and MMP-1 expression are not always inversely regulated, as collagen-synthetic pathways can be activated independently [30]. Further investigation of the TGF-β/Smad axis [31] would help clarify the mechanism by which compound **1** promotes collagen synthesis in fibroblasts.

Compound **1** also significantly inhibited TNF-α-induced JNK phosphorylation, a key MAPK driving MMP-1 expression [32,33,34], supporting its anti-aging efficacy. Moreover, compound **1** exhibited potent anti-inflammatory activity by suppressing TNF-α-induced IL-6, IL-8, and IL-1β expression at low to moderate concentrations [35]. Its broad anti-inflammatory effects, previously attributed to NF-κB inhibition and MAPK attenuation, are consistent with our findings. However, at 100 μM, both IL-6 and IL-8 showed a modest rebound, reflecting the known biphasic response of polyphenols, in which high concentrations shift toward pro-oxidant behavior and mildly reactivate NF-κB signaling [36].

Reducing inflammatory cytokine levels is particularly important in dermal fibroblasts, as chronic inflammation disrupts ECM remodeling and accelerates photoaging. By mitigating ROS and inhibiting MAPK and NF-κB activation, compound **1** interrupts the feed-forward cycle of oxidative and inflammatory damage, supporting its potential for both anti-aging and inflammation-related dermatological applications. Although a slight cytokine rebound indicates dose-dependent effects, it underscores the need for optimal concentration ranges and comprehensive toxicity assessments [37].

Recent reports suggest that compound **1** may also enhance skin barrier-related gene expression, including filaggrin and involucrin [38], and modulate additional aging-related pathways, such as Nrf2 and AMPK [39]. Evaluating these pathways in TNF-α-stimulated NHDFs would deepen our understanding of their regulatory roles. Given that compound **1** activates the Nrf2/ARE axis and promotes endogenous antioxidant enzymes [38], further analysis could clarify its contribution to redox homeostasis.

Overall, our results emphasize the multifunctional protective effects of *P. japonicum* compounds, with compound **1** showing particularly strong activity in mitigating oxidative stress, preserving extracellular matrix integrity, reducing inflammation, and promoting collagen synthesis. These results highlight *P. japonicum* as a promising natural ingredient for skincare formulations aimed at preserving skin structure and function.

To further substantiate these results, future studies employing three-dimensional (3D) skin models or in vivo photoaging systems will be important to confirm the efficacy of these compounds under more physiologically relevant conditions. Furthermore, evaluating the skin permeability, formulation stability, delivery optimization, and long-term safety of the compound is critical for translational development. Given the complex phytochemical composition of *P. japonicum*, investigating potential synergistic or additive interactions among its constituents may further enhance its anti-aging efficacy and support the development of multi-target natural dermal formulations.

## 5. Conclusions

This study demonstrated that *P. japonicum* extracts and isolated compounds exert notable aging- and inflammatory-modulating effects on TNF-α-stimulated human dermal fibroblasts (an inflammatory cytokine associated with skin aging). Among the six compounds tested, compound **1** demonstrated the strongest biological activity, marked by a significant reduction in intracellular ROS levels, inhibition of MMP-1 expression, and promotion of the biosynthesis of type I collagen, a structural protein crucial for maintaining skin integrity and elasticity. Additionally, compound **1** inhibited the phosphorylation of JNK, thereby reducing MMP expression and contributing to the preservation of the extracellular matrix. In addition to its antioxidant properties, compound **1** significantly suppressed the mRNA expression of major inflammatory cytokines such as IL-6, IL-8, and IL-1β, highlighting its potent anti-inflammatory potential. These findings identify compound **1** as the major active component responsible for the dual antioxidant and anti-inflammatory effects of *P. japonicum*. Through coordinated modulation of pathways involved in structural breakdown and inflammatory responses, it shows promise as a valuable natural ingredient for cosmetic strategies that protect the skin from inflammation-related damage. Future research should focus on improving its bioavailability and evaluating efficacy in advanced skin models to support its clinical and commercial development.

## Figures and Tables

**Table 1:** Quantitative HPLC analysis of six major phytochemicals in *P. japonicum* extracts.

Extract	Content (mg/g Extract)						
	1	2	3	4	5	6	Total
YML	29.98 ± 0.18	1.30 ± 0.00	7.01 ± 0.04	1.36 ± 0.01	6.89 ± 0.03	12.81 ± 0.06	59.35
YMR	1.08 ± 0.01	7.22 ± 0.06	11.01 ± 0.10	8.50 ± 0.06	13.85 ± 0.10	8.14 ± 0.06	49.80
YAL	28.52 ± 0.66	1.84 ± 0.05	9.19 ± 0.24	1.42 ± 0.04	7.14 ± 0.19	12.85 ± 0.34	60.96
YAR	1.38 ± 0.00	7.83 ± 0.03	15.60 ± 0.06	11.65 ± 0.04	7.70 ± 9.58	10.75 ± 0.04	54.91
YNL	18.07 ± 0.19	3.17 ± 0.05	7.50 ± 0.12	3.54 ± 0.86	8.81 ± 2.03	10.06 ± 0.15	51.15
YNR	0.44 ± 0.00	6.63 ± 0.05	7.64 ± 0.06	12.62 ± 0.17	14.26 ± 0.12	5.67 ± 0.04	47.26
YML	29.98 ± 0.18	1.30 ± 0.00	7.01 ± 0.04	1.36 ± 0.01	6.89 ± 0.03	12.81 ± 0.06	59.35

## Data Availability

The original contributions presented in this study are included in the article. Further inquiries can be directed to the corresponding authors.
